# A Cryptic *Frizzled* Module in Cell Surface Collagen 18 Inhibits Wnt/β−Catenin Signaling

**DOI:** 10.1371/journal.pone.0001878

**Published:** 2008-04-02

**Authors:** Delphine Quélard, Elise Lavergne, Ismaïl Hendaoui, Harri Elamaa, Ulla Tiirola, Ritva Heljasvaara, Taina Pihlajaniemi, Bruno Clément, Orlando Musso

**Affiliations:** 1 INSERM, U620, University of Rennes-1, Rennes, France; 2 Biocenter, Department of Medical Biochemistry and Molecular Biology, University of Oulu, Oulu, Finland; Stanford University, United States of America

## Abstract

Collagens contain cryptic polypeptide modules that regulate major cell functions, such as cell proliferation or death. Collagen XVIII (C18) exists as three amino terminal end variants with specific amino terminal polypeptide modules. We investigated the function of the variant 3 of C18 (V3C18) containing a *frizzled* module (FZC18), which carries structural identity with the extracellular cysteine-rich domain of the *frizzled* receptors. We show that V3C18 is a cell surface heparan sulfate proteoglycan, its topology being mediated by the FZC18 module. V3C18 mRNA was expressed at low levels in 21 normal adult human tissues. Its expression was up-regulated in fibrogenesis and in small well-differentiated liver tumors, but decreased in advanced human liver cancers. Low FZC18 immunostaining in liver cancer nodules correlated with markers of high Wnt/β−catenin activity. V3C18 (M_r_ = 170 kD) was proteolytically processed into a cell surface FZC18-containing 50 kD glycoprotein precursor that bound Wnt3a *in vitro* through FZC18 and suppressed Wnt3a-induced stabilization of β−catenin. Ectopic expression of either FZC18 (35 kD) or its 50 kD precursor inhibited Wnt/β−catenin signaling in colorectal and liver cancer cell lines, thus downregulating major cell cycle checkpoint gatekeepers cyclin D1 and *c-myc* and reducing tumor cell growth. By contrast, full-length V3C18 was unable to inhibit Wnt signaling. In summary, we identified a cell-surface signaling pathway whereby FZC18 inhibits Wnt/β−catenin signaling. The signal, encrypted within cell-surface C18, is released by enzymatic processing as an active *frizzled* cysteine-rich domain (CRD) that reduces cancer cell growth. Thus, extracellular matrix controls Wnt signaling through a collagen-embedded CRD behaving as a cell-surface sensor of proteolysis, conveying feedback cues to control cancer cell fate.

## Introduction

The cell surface matrix is a signaling platform integrating bidirectional cues between cells and extracellular matrix (ECM), which contains growth factors, proteases and bioactive polypeptides, thus controlling major signaling pathways. Cryptic bioactive polypeptides become functional by structural or conformational changes of parent ECM molecules, including proteolysis [1].

Collagen XVIII (C18) [2], [3], the parent molecule of endostatin [4], is expressed as three distinct variants by two separate promoters and alternative splicing of one of the transcripts [3], [5]. Promoter #1 generates variant #1 (V1C18), which is a ubiquitous structural basement membrane component. Alternative splicing of transcripts from promoter #2 generates variants #2 (V2C18) and #3 (V3C18) [6], [7], which are secreted under the control of both liver-specific and ubiquitous transcription factors. V2C18 contains a 192-aa Domain of Unknown Function-959 (DUF-959) at its N-terminus, followed by sequences common to all variants [8]. V2C18 is secreted as a plasma protein by hepatocytes [9] and its expression decreases in advanced liver cancers [10]. V3C18 carries a 235-aa stretch with 10 conserved cysteines, bearing sequence and structural identities with the extracellular cysteine-rich domain (CRD) of the *frizzled* (FZ) receptors and the secreted *frizzled*-related proteins (SFRPs) [11]. Hence, we named this module FZC18.

In the Wnt/β−catenin signaling pathway, interaction of 10 different FZ cell-surface receptors with at least 19 Wnts can be inhibited by a family of 5 SFRPs that bind to Wnts at the cell surface. In the absence of Wnt, β−catenin is recruited into a destruction complex, phosphorylated at conserved N-terminal residues by GSK3β, and thus tagged for proteasomal degradation [12]. In the nucleus, target genes of the pathway are repressed by co-repressor binding to T-cell factor (TCF) transcription factors. Wnt binding to FZ receptors blocks β-catenin phosphorylation, which becomes resistant to proteasomal degradation. Consequently, β-catenin accumulates both in the cytoplasm and the nucleus, displacing co-repressors from TCF and activating transcription of target genes that regulate the balance between proliferation and apoptosis, differentiation and metabolism, including cyclin D1, *c-myc* [12] and glutamine synthetase (GS) [13].

Wnt/β−catenin signaling can be activated in human cancers by oncogenic mutations or by epigenetic silencing of pathway components. Respectively, 85% and 10% of sporadic colorectal cancers (CRCs) have APC and β−catenin mutations [12]. Epigenetic inactivation of SFRP genes occurs early in CRC [14] and restoring SFRP expression attenuates Wnt/β−catenin signaling [15]. In 30–40% of human hepatocellular carcinomas (HCCs), activation of the pathway results from β−catenin and axin mutations [16] or epigenetic silencing of SFRP1 [17].

We investigated whether FZC18 could regulate Wnt/β−catenin signaling. We show that V3C18 mRNA expression decreases in advanced HCCs. In addition, the N-terminus of the V3C18 is partially processed to a FZC18 precursor (V3Nter) which pulls down Wnt3a *in vitro*, suppresses Wnt/β−catenin signaling and downstream gene expression, reduces tumor cell growth and increases cell death in CRC and HCC cell lines carrying activating β−catenin mutations. The biological activity of FZC18 is cryptic within full-length C18, as it is activated by proteolytic release.

## Results

### Decreased expression of V3C18 in cancer cell lines and in advanced primary liver cancers

**Figure 1:**
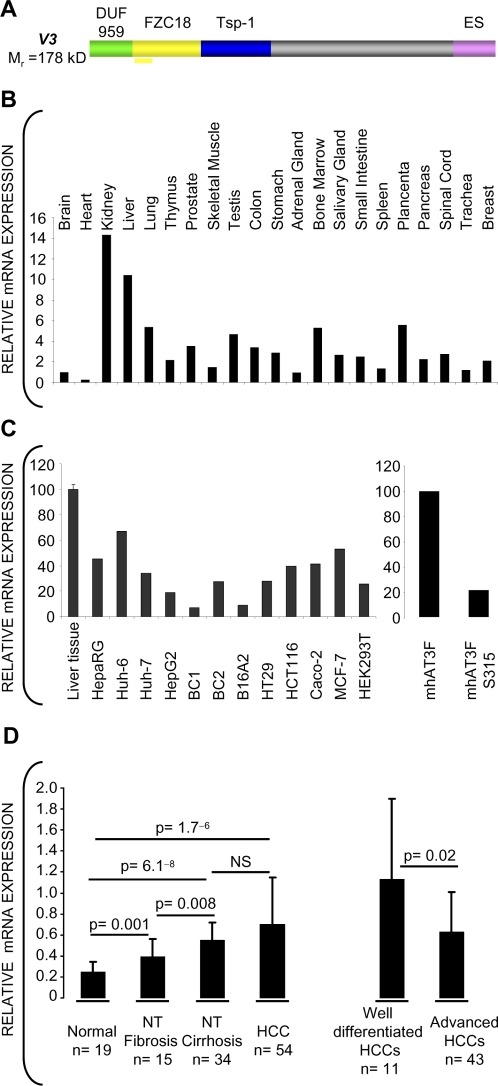
Reduced expression of FZC18 in advanced liver cancers and cell lines. (A) Schematic of V3C18 showing DUF-959, FZC18, Tsp-1 (thrombospondin-1) and ES (endostatin) modules. The thick horizontal yellow line below FZC18 indicates the probe used. (B–D) Relative mRNA expression of V3C18 by QRT-PCR in human tissues (Human Total RNA Master Panel, Clontech, USA) (B) and in cell lines (C). Assays were performed in triplicate with a coefficient of variation <10% and normalized to 18S. Brain (B) and liver (C) were used as calibrators. (D) Relative V3 mRNA expression in human liver samples. Small (≤2 cm), well-differentiated HCCs are compared to advanced HCCs. mRNA samples were blotted in triplicates onto nylon membranes and arrays hybridized with ^32^P-labeled cDNA under linear-range conditions. Densitometry readings were normalized with an 18S probe. Bar graphs show mean±SD. The Mann Whitney's “U” test was used. NS, non significant; NT, non tumor livers.

We analyzed the mRNA levels of V3C18 in human tissues by QRT-PCR (quantitative RT-PCR) using brain as a calibrator (Figure 1B). V3C18 showed the highest levels in kidney, followed by liver, placenta, lung, bone marrow and testis. The expression of V3C18 was 2 to 14 folds lower in cell lines from human HCC, CRC, breast cancer or embryonic kidney than in normal liver (Figure 1C). In addition, in the well-differentiated mouse hepatoma cell line mhAT3FS315, carrying a dominant negative form of the HNF3 transcription factor, inhibiting expression of genes controlling the hepatocyte phenotype [18], the expression of V3C18 mRNA was 22% of that in the parental cell line mhAT3F (Figure 1C).

Low expression of V2C18 is associated with tumor progression and reduced disease-free survival in HCCs [10]. Although V2C18 and V3C18 share a common promoter, V3C18 is additionally regulated by alternative splicing of FZC18 to produce V2 [7]. Analysis of mRNA arrays from 122 frozen liver samples included normal livers from 19 subjects, 54 HCCs and 49 matching non tumor livers. V3C18 mRNA levels were higher in fibrotic and cirrhotic livers than in normal livers (Figure 1D), indicating that the expression of V3C18 increases during tissue remodeling. Small (≤2 cm) well-differentiated HCCs showed higher V3C18 mRNA levels than advanced HCCs. The mean±SD size of both groups was 1.3±0.38 cm and 6.5±4.6 cm, respectively (p = 3^−07^). As previously shown for V2C18 [10], these findings indicate that higher V3C18 mRNA expression may be associated with less aggressive tumors.

### V3C18 and its amino-terminal proteolyzed form *V3Nter* localize at the cell surface through the FZC18 module

**Figure 2:**
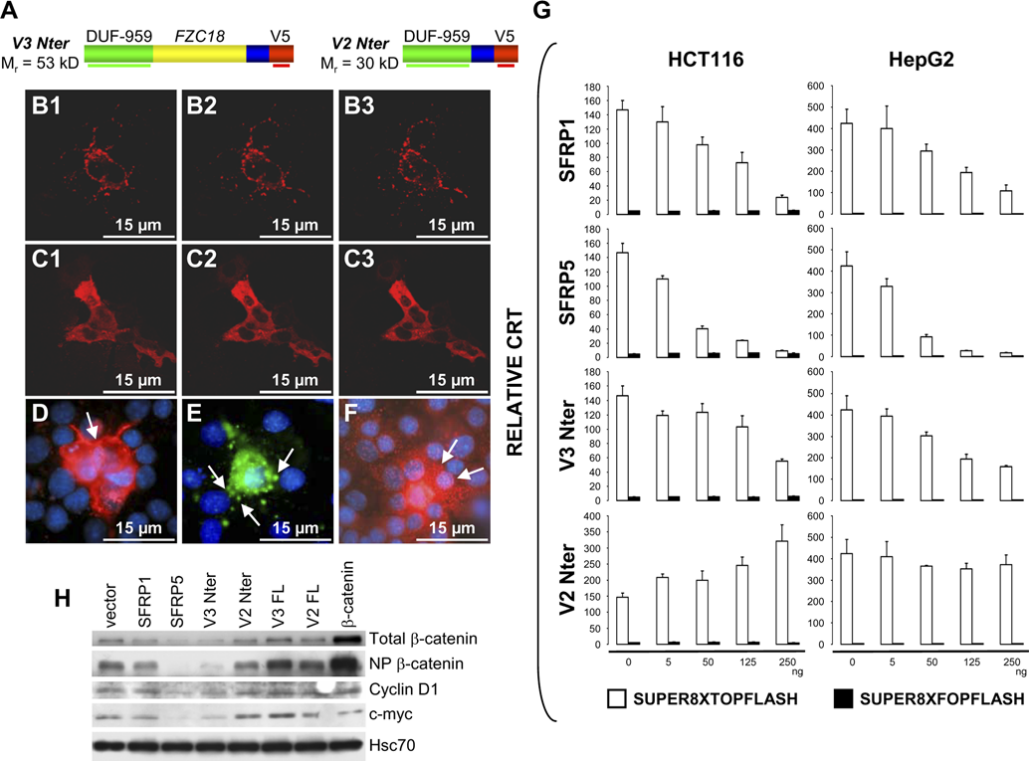
V3Nter localizes at the cell surface and inhibits Wnt/β−catenin signaling and downstream protein expression. (A) V3Nter and V2Nter expression vectors. Thick horizontal color lines indicate the antibodies used. Blue box, 47-aa stretch from the Tsp-1C18 module. (B1-C3) Confocal fluorescence microscopy of mhAT3FS315 mouse HCC cells transiently transfected with V3Nter (B1-B3) or V2Nter (C1-C3) cDNAs and probed with anti-DUF-959, followed by anti-rabbit TRITC-labeled IgG *(red).* Three out of 15 representative images acquired at 500-nm intervals on the vertical axis are shown. V3Nter (B1-B3) highlights intercellular spaces. V2Nter (C1-C3) is detected in the cytoplasm. Movie S1 shows a tridimensional reconstruction of V3Nter cell surface staining using the 15 acquired images (original magnification x1000). Figure S1, B-D shows confocal microscopy of V3FL and V2FL. (D-F) Fluorescence microscopy of mouse HCC cells transfected with either V3Nter (D and E) or V2Nter (F) cDNAs and incubated with either anti-DUF-959, followed by anti-rabbit TRITC-labeled IgG *(red)* (D and F) or anti-V5 epitope tag, followed by anti-mouse FITC-labeled IgG (E). (D) V3Nter outlines cell membranes and cell-cell boundaries *(arrow).* (E) V3Nter highlights the cell surface *(arrows).* (F) Secretion of V2Nter covers the cell surface and is detected on neighboring cells as dispersing red speckles *(arrows).* (G) Dose-dependent changes in CRT in response to increasing amounts of transiently transfected cDNA vectors. Reporter gene assays using a β−catenin-TCF reporter driven by wild-type (*SUPER8XTOPFLASH,* white bars) or a negative control with mutated TCF binding sites (*SUPER8XFOPFLASH,* black bars). Results are means of three replicates from a representative experiment. Three independent experiments were performed. Error bars represent standard deviations. Figure S1, E and F shows changes in CRT in response to V3FL and V2FL. (H) Immunoblot of HCT116 cells transiently transfected and probed with the indicated cDNA vectors *(top)* and antibodies *(right)*. Hsc70 is a loading standard.

Human embryonic kidney cells secrete a ∼45 kD amino-terminal fragment of V3C18 containing the FZC18 module [7]. These data led us to construct an expression vector including the natural signal peptide+DUF-959+FZC18 modules and 47 aa from the Tsp-1C18 domain common to all C18 variants that we called *V3Nter* (Figure 2A). As a control, *V2Nter* included the same sequences, but lacked FZC18 (Figure 2A).

Transient transfection of V3Nter or V2Nter cDNAs in mhAT3FS315 mouse hepatoma cells followed by immunocytochemistry (Figure 2, B–F) showed that V3Nter highlighted cell-cell boundaries (Figure 2, B, D and Movie S1) and cell membranes, particularly at sites of cell-cell contact (Figure 2D), as well as the cell surface (Figure 2E). By contrast, V2Nter was detected within the cells (Figure 2C). The surface of some of the cells was covered with V2Nter signal, with dispersing speckles detected on neighboring cells (Figure 2F), suggesting secretion.

Transient transfection of full-length V3C18 (V3FL) or full-length V2C18 (V2FL) cDNAs in mouse hepatoma cells followed by confocal immunofluorescence confirmed the above findings. V3FL outlined cell membranes (Figure S1B), whereas V2FL was detected within the cell. Cell membranes and intercellular boundaries showed no signal (Figure S1C). V2FL was also observed within large intracellular vacuoles resembling Golgi structures (Figure S1D). The cell surface showed fraying vesicles positive for V2FL, suggesting secretion (Figure S1D). Strong intracellular burden of V2Nter and V2FL in cells transiently expressing these proteins at high levels is consistent with our previous observation of intracellular signal for endogenous V2C18 in hepatocytes *in vivo* and is frequently observed in the case of other abundantly secreted plasma or ECM proteins such as albumin or fibronectin [9], [10], [19]. In addition, abundant intracellular material in some of the cells expressing V2C18 or V3C18 *in vitro* and *in vivo* suggested posttranslational maturation of these proteins *(i.e.,* glycosylation, see below).

### V3Nter is a cryptic inhibitor of Wnt/β−catenin signaling

SFRP1, SFRP2 and SFRP5 suppress β−catenin−T−cell factor (TCF)-regulated transcription (CRT) from a TCF/LEF responsive reporter in the CRC cell line HCT116 [15]. We asked whether V3Nter and V3FL could inhibit Wnt/β−catenin signaling in cell lines carrying activating β−catenin mutations, HCT116 (β−catenin ΔS45) and HepG2 (HCC, β−catenin Δ25−140) and used SFRP1 and SFRP5 as controls. Ectopic expression of SFRP1, SFRP5 or V3Nter suppressed CRT in a dose-dependent manner (Figure 2G). By contrast, V2Nter (Figure 2G), V2FL and V3FL (Figure S1E), increased CRT in HCT116, but not in HepG2 cells. The increase in CRT by V2Nter, V2FL and V3FL was also observed in other cell lines. In the well-characterized human HCC cell line Huh-7 (wild-type β-catenin, baseline Wnt/β−catenin signaling [20]) Wnt1, V2FL and V3FL increased CRT by more than 10 folds and V2Nter by 2.9 folds (Figure S1F). Similar results were obtained in the mhAT3FS315 mouse HCC cell line (not shown).

C18 is a heparan sulfate proteoglycan (HSPG), which contains heparan sulfate attachment sites common to all variants [8]. Thus, endogenous V2C18 and V3C18, as well as their full-length and their amino-terminal constructs could be HSPGs. Consistently, immunoblotting of proteoglycan-enriched fractions from a human HCC with anti-FZC18, anti-DUF-959 and anti-Tsp-1C18 antibodies showed a high molecular weight smear typical of HSPGs. Important mobility shift and epitope unmasking were seen after digestion with heparin lyases, but not with chondroitinase ABC (Figure S1, A and G). Transfection of V2FL and V3FL in mouse hepatoma cells and detection of the most N-terminal (DUF-959) and C-terminal (endostatin) epitopes, as well as FZC18, confirmed the high molecular weight smear typical of HSPG for V2FL and V3FL (Figure S1H). Indeed, V2FL was detected in cell-conditioned medium (Figure S1H). By contrast, V3FL was detected at high levels in cell layers but not in the conditioned medium (Figure S1H), confirming its predominantly cell-surface location (Figure S1B). Therefore, V2FL-HSPG and V3FL-HSPG might locally increase Wnt concentration, thereby increasing CRT activity, as has been shown for other HSPGs [21]. In addition, V2Nter and V3Nter share a C-terminal 47 amino-acid stretch (Figure 2A) from the heparan sulfate attachment site of C18 [8], probably explaining the slight increase in CRT by V2Nter (Figure 2G and S1F).

Transient overexpression of V3Nter resulted in a reduced protein content of total β-catenin, non phosphorylated β-catenin, *c-myc* and cyclin D1 in HCT116 cells (Figure 2H). Consistently with data on CRT, V2FL and V3FL did not reduce the protein content of total and non phosphorylated β−catenin, cyclin D1 or *c-myc* (Figure 2H). In addition, consistently with decreased cyclin D1 protein expression, reporter gene assays using the cyclin D1 promoter [22] upstream of luciferase cDNA confirmed that SFRP1, SFRP5 and V3Nter decreased cyclin D1 promoter activity in HCT116 and HepG2 cells (Figure S1I). Remarkably, overexpression of β-catenin increased cyclin D1 promoter activity by 2.4 and 4.8 folds in HCT116 and HepG2 cell lines, respectively. Taken together, these data show that V3Nter can inhibit β-catenin signaling in cancer cells carrying activating β-catenin mutations and that the biological activity of the *frizzled* CRD is cryptic within full-length cell surface C18-HSPG.

### V3Nter inhibits tumor cell growth through increased cell death

**Figure 3:**
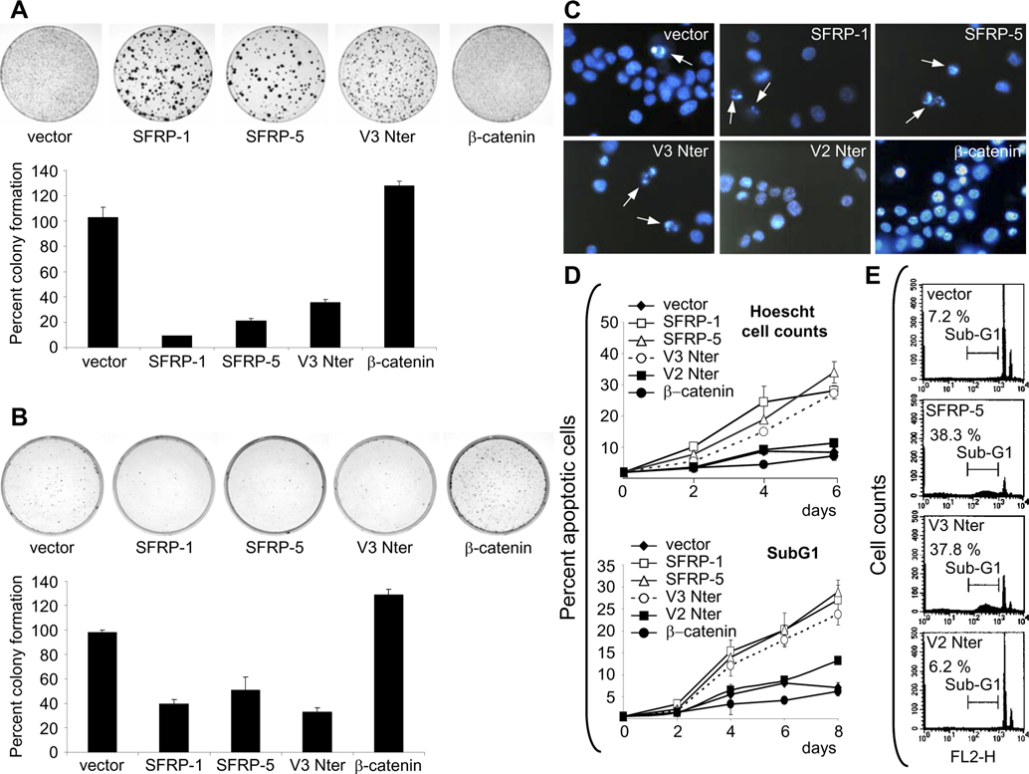
V3Nter decreases colony formation and induces tumor cell death in HCT116 and HepG2 cells. HCT116 (A, C and D) and HepG2 (B, E) cells were transfected with cDNA vectors and selected for 14 d (A and B), 4 d (C and E) or 6–8 d (D) with G418. (A and B) After hematoxylin staining, colonies (seen as dark spots) were digitized using a video camera and counted with Scion Image (NIH). Histograms show colony formation efficiencies relative to cells transfected with empty vector. (C) Hoescht 33342-stained cells photographed at ×200 magnification. Arrows indicate apoptotic cells. (D) Hoescht cell counts show mean±SD percent apoptotic cells out of triplicate 300-cell counts from 6-well plates (blindly at ×200 magnification) at each time point. SubG1 shows mean±SD percent apoptotic cells out of triplicate 1×10^4^ cells from 6-well plates assessed by flow cytometry at each time point. (E) Representative FL2-H histograms of HepG2 cells show percent SubG1 population.

Based on these findings, we analyzed the effects of V3Nter on growth and death of tumor cells. Decreased colony formation occurred in HCT116 and HepG2 cells overexpressing V3Nter. Inhibition of clonogenesis by V3Nter, SFRP1 and SFRP5 were within the same range in both cell lines (Figure 3, A and B). β-catenin induced a ∼30 % increase in colony formation in HCT116 and HepG2 cells, in consistency with data on cyclin D1 promoter activity, thus supporting the hypothesis that despite activating β-catenin mutations, cells can still respond to stimuli inducing further increases in β-catenin levels. HCT116 cells overexpressing SFRP1, SFRP5 or V3Nter showed chromatin condensation, nuclear fragmentation, numerous apoptotic bodies and an overall low cell density. By contrast, higher cell densities and rare apoptotic bodies were seen in cells expressing V2Nter, β-catenin or vector alone (Figure 3C). Numbers of morphologically apoptotic Hoescht-stained cells and flow cytometry analysis of subG1 cells showed that V3Nter induced tumor cell death within the same range as SFRPs (25 to 35%) in HCT116 cells (Figure 3D). Similarly, SubG1 analysis on HepG2 cells showed 35–40% cell death (Figure 3E). As expected, vector alone and V2Nter showed baseline levels of cell death in HCT116 and HepG2 cells (Figure 3, D and E).

### Amino terminal processing of V3Nter releases soluble FZC18

**Figure 4:**
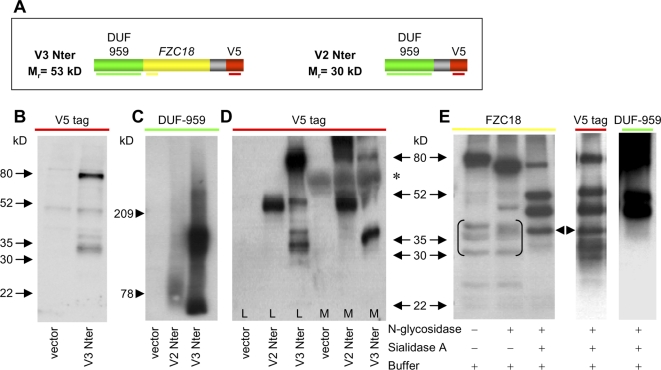
Processing of V3Nter releases FZC18. (A) Schematic of V3Nter *(left)* and V2Nter *(right)* cDNA vectors. Horizontal color lines below each module indicate the antibodies used. (B) Immunoblot of protein extracts from HCT116 cells transfected with the indicated cDNAs, resolved in denaturing 7.5% PAGE-SDS and probed with anti-V5 *(red).* V3Nter shows ∼80 kD and ∼30–40 kD polypeptides. (C–E) Protein extracts from mhAT3FS315 cells transfected with the indicated cDNAs. Denaturing 5% (C) or 7.5% (D–E) PAGE-SDS gels. (C) V2Nter and V3Nter appear as smears typical of proteoglycans. (D) *Asterisk,* immunoglobulins from medium containing FCS. V2Nter shows a ∼50 kD polypeptide in cell layers *(L)* and conditioned medium *(M).* V3Nter shows ∼80 kD and ∼30–40 kD polypeptides in cell layers *(L).* By contrast, in medium *(M),* a 36 kD polypeptide predominates and the 80 kD form decreases in intensity. Note that 5 µg of protein was loaded for cell layers and 50 µg for conditioned media. The ∼80-kD V3Nter polypeptide is detected at much higher levels in cell layers than in media. In addition, V2Nter polypeptides are detected as a high molecular weight smear above the 80 kD marker in conditioned medium. (E) mhAT3FS315 cells transfected with V3Nter cDNA. Cell extracts incubated at 37°C for 2 hr with N-glycosidase, Sialidase A or buffer alone and probed with the indicated antibodies. Deglycosylation reveals polypeptides of ∼50 and 48 kD detected by anti-FZC18, anti-V5 and anti-DUF-959. A 36 kD polypeptide *(arrowheads)* is detected by anti-FZC18, anti-V5, but not by anti-DUF-959. [Anti-FZC18 detects nonspecific polypeptides between 30–40 kD *(brackets)*, consistently with Figure 8G].

HCT116 cell layers expressing V3Nter (M_r_ = 53 kD, Figure 4A) showed 80 kD and ∼30–40 kD polypeptides (Figure 4B). We thus studied the relevance of glycosylation and further proteolysis of bioactive V3Nter in the mouse HCC cell line mhAT3FS315. This cell line expresses endogenous V3C18 at very low levels (Figure 1C), which is not detected by anti-human FZC18 antibody (not shown). Figure 4C shows that V2Nter (M_r_ = 30 kD) and V3Nter migrated as high molecular weight smears typical of proteoglycans, consistently with data shown in Figure S1, G and H. This is explained by heparan sulfate attachment sites residing within the 47-aa stretch common to all variants [8] present in V2Nter and V3Nter constructs. Therefore, as has been shown for other HSPGs [21], V2Nter may locally increase Wnt concentration, resulting in a slight increase in CRT. However, the strong inhibition of Wnt/β−catenin signaling by V3Nter could offset the slight increase in CRT by this 47 amino-acid stretch.

Analysis of mhAT3FS315 cell layers confirmed the 80 kD and ∼30–40 kD FZC18-containing polypeptides detected in HCT116 cells (Figure 4D). Consistently with its predominant cell surface topology, the ∼80-kD V3Nter polypeptide was detected at much lower levels in cell conditioned media than in cell layers, even after loading a 10-fold higher amount of protein from conditioned media than from cell layers (Figure 4D). Similarly, V3FL was not detected in the conditioned medium (Figure S1H), suggesting that it is fully deposited within the cell surface matrix. Indeed, V3Nter is a noncollagenous domain, whereas V3FL contains both noncollagenous and collagenous domains. Thus, V3FL may be secreted as a triple helical homotrimer, being deposited in the pericellular matrix through supramolecular assemblies with other extracellular matrix molecules, such as laminins or cell surface heparan sulfates, which bind C18 through the endostatin domain [23]. In addition, V3FL may bind cell surface integrin receptors through RGD sequences in the collagenous domains [2]. By contrast, conditioned media showed a higher ratio of the 36 kD/80 kD FZC18-containing polypeptides than cell layers (Figure 4D, compare V3Nter in medium and in cell layers), suggesting that proteolytic processing releases a soluble FZC18 polypeptide.

Digestion of V3Nter with N-glycosidase and Sialidase A (Figure 4E) revealed ∼50 and 48 kD polypeptides containing FZC18, V5 and DUF-959 epitopes. In addition, a 36 kD polypeptide contained FZC18 and V5, but not DUF-959, suggesting that N-terminal processing of V3Nter results in the release of FZC18. To test whether FZC18 alone was active, we cloned the FZC18 module in frame with the IgΚ signal sequence and a C-terminal *c-myc* tag (Figure S2A). Expression in mhAT3FS315 cells revealed a soluble ∼35 kD N-glycosylated polypeptide in cell conditioned medium (Figure S2B). The close correspondence between the calculated (M_r_ = 31 kD) and the experimentally detected (∼35 kD) molecular mass of FZC18 suggested that sialylation may not reside within FZC18. Consistently, Sialidase A digestion of V2Nter showed a ∼20 kD mobility shift (Figure S2C). Thus, sialic acid moieties in V3Nter may be responsible for FZC18 epitope masking. Consistently with the above findings, sialic acid is known to target glycoproteins to the extracellular side of the plasma membrane [24] and plays an important role in cell surface recognition and signaling.

### FZC18 suppresses Wnt/β−catenin signaling

**Figure 5:**
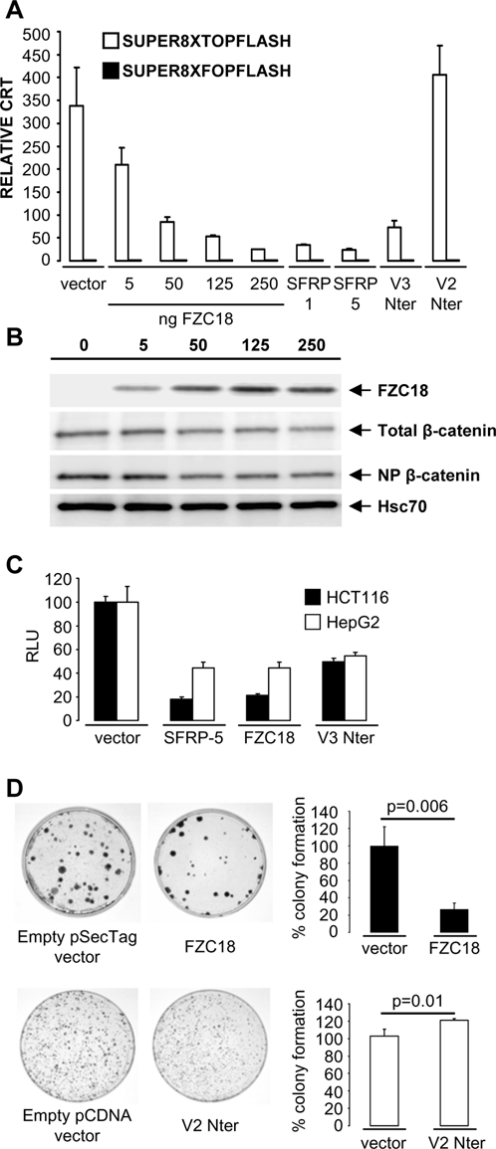
FZC18 suppresses Wnt/β−catenin signaling and clonogenesis in cancer cells. (A) Reporter gene assays using a β−catenin-TCF responsive reporter *(SUPER8XTOPFLASH*, white bars) and a negative control *(SUPER8XFOPFLASH*, black bars) in HCT116 cells. Dose-dependent decrease in CRT is detected in response to increasing amounts of transiently transfected FZC18 cDNA. Controls include SFRP1, SFRP5, V3Nter and V2Nter (250 ng cDNA). (B) Immunoblot of HCT116 cells transiently transfected with increasing amounts of FZC18 cDNA. The blots were probed with the indicated antibodies *(right).* Hsc70 is a loading standard. (C) Reporter gene assays using cyclin D1 promoter driving luciferase expression in transiently transfected HCT116 and HepG2 cells. Results are expressed relative to cells transfected with empty vector. (D) Colony formation assay in HCT116 cells transfected with FZC18 or V2Nter. Histograms show colony formation efficiencies.

FZC18 suppressed CRT and total and non-phosphorylated β−catenin stabilization in a dose-dependent manner (Figure 5, A and B). In addition, FZC18 downregulated cyclin D1 promoter activity (Figure 5C) and decreased colony formation (Figure 5D) by 75% compared with cells expressing vector alone. In contrast, V2Nter induced a 21% increase in colony formation with respect to empty vector (Figure 5D), consistently with data on CRT (Figures 2G and S1F) and cyclin D1 promoter activity (Figure S1I).

### Ectopic FZC18 binds Wnt3a and suppresses Wnt3a-dependent activation of β−catenin signaling *in vitro*

**Figure 6:**
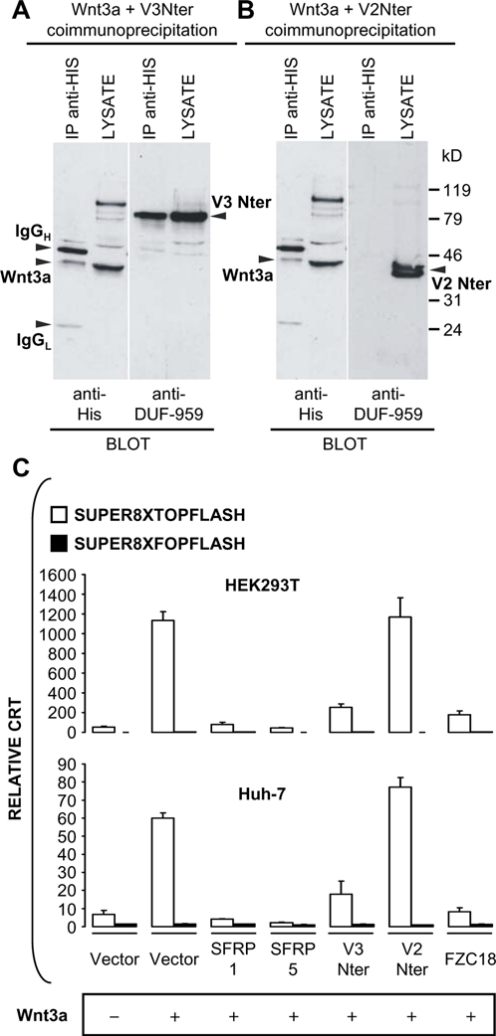
Ectopic FZC18 binds Wnt3a and suppresses Wnt3a-dependent activation of β−catenin signaling *in vitro.* (A and B) Wnt3a pulls down V3Nter specifically via the FZC18 domain. EBNA-293 cells were cotransfected with V3Nter (A) or V2Nter (B) and His-tagged mouse Wnt3a. Cell lysates were immunoprecipitated *(IP)*, resolved by 10% PAGE-SDS and immunoblotted with the indicated antibodies. IgG_H_ and IgG_L_ are immunoglobulin heavy and light chains. (C) Reporter gene assays using a β−catenin-TCF responsive reporter *(SUPER8XTOPFLASH)* or a negative control *(SUPER8XFOPFLASH).* HEK293T or Huh-7 cells were cotransfected with Wnt3a and the indicated vectors. Results are means of three replicates from a representative experiment. Three independent experiments were performed. Error bars represent standard deviations.

EBNA-293 cells were cotransfected with His-tagged Wnt3a and either mouse V3Nter or V2Nter expression vectors. Wnt3a pulled down V3Nter but not V2Nter (Figure 6, A and B), as shown by immunoblotting with anti-DUF-959 antibody. In addition, previous incubation of transfected cells with increasing concentrations of a 15-aa peptide derived from the CRD of FZC18 competed with FZC18 pull-down by Wnt3a (Figure S3A), suggesting that Wnt3a interacts directly with the CRD of FZC18. Then, we searched for *in silico* models predicting the 3D structure of the FZC18 CRD using threading algorithms that seek for template proteins with well-characterized crystal structures in PDB databases *(Phyre* www server). Two highly significant matches were mouse SFRP3 and FZ8, showing 32% and 22% identity, respectively. E-values were 1.4^−15^ for SFRP3 and 6.6^−15^ for FZ8, with an estimated precision of 100% for both models. Similar results were obtained by homology modeling using the www server HHpred, indicating 100% probability that the predicted 3D model of FZC18 CRD matches the templates SFRP3 and FZ8 (p = 0). Next, we looked at the localization of the competing peptide RH3 on the putative surface of the FZC18 CRD. Running the above models on Protein Explorer 2.79 showed that RH3 lies at the FZC18 CRD solvent-exposed surface (Figure S3B). In addition, the competing peptide lies adjacent to and partially overlaps residues involved in Wnt-mFZ8 CRD interactions [25]. Consistently, cotransfection of mouse Wnt3a and human FZC18 or V3Nter with a TCF-responsive reporter in the HEK293T and in the Huh-7 cell lines showed that FZC18 and V3Nter suppressed Wnt3a-induced CRT by more than 80% (Figure 6C). Taken together, these data show that FZC18 may interact with Wnt3a, suggesting that FZC18 may function as a SFRP-like bioactive polypeptide quenching at least Wnt3a in the tumor microenvironment.

### Negative correlation between FZC18 expression and β−catenin pathway activity *in vivo*

**Figure 7:**
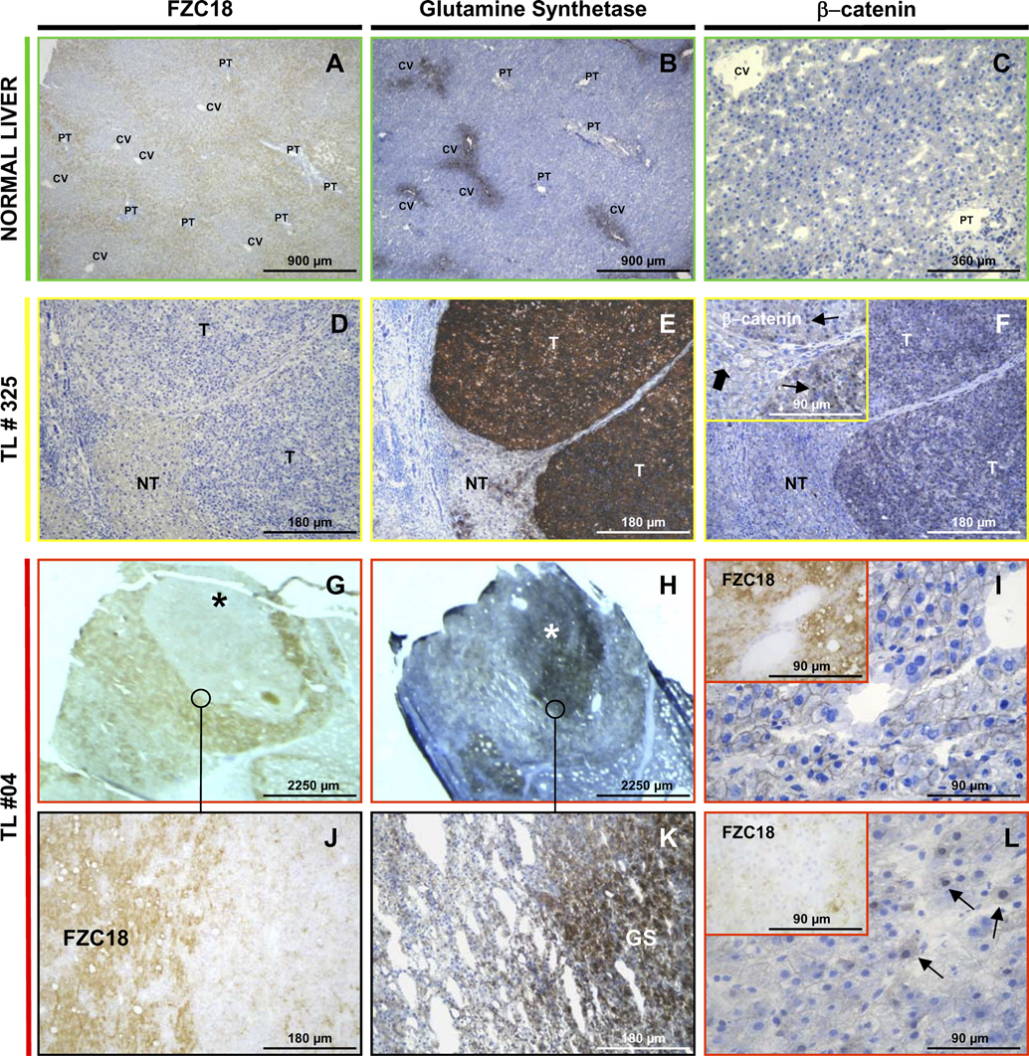
FZC18 is negatively associated with Wnt/β−catenin pathway activity *in vivo.* Immunoperoxidase detection *(brown)* of FZC18, glutamine synthetase (GS) or β−catenin in normal and tumor livers. Hematoxylin counter-staining *(blue).* (A-C) In normal liver, FZC18 (A) is detected around portal tracts *(PT).* No FZC18 is seen around central veins *(CV).* GS (B) is detected around CV. (C) Faint cell-membrane β−catenin. (D–F) Contiguous sections of tumor liver tissue (TL #325) arising in a cirrhotic nodule. FZC18 (D) is detected in remaining non tumor hepatocytes *(NT),* compressed by the expansive growth of the tumor, but not in the tumor *(T).* GS (E) is strong in *T* and faint in *NT*. β−catenin (F) is detected in cell membranes in *NT (thick arrow)* and in cytoplasm and nuclei in *T (thin arrows) (inset).* (G–L) Contiguous sections of tumor liver tissue (TL #04). Nodule-in-nodule showing faint FZC18 (G), but strong GS (H) staining *(asterisks),* surrounded by tumor tissue showing strong FZC18, but faint GS staining. (J and K) Higher magnification. (I) Strong FZC18 staining *(inset),* associated with membranous β−catenin. (L) Mild FZC18 staining *(inset),* associated with cytoplasmic and nuclear β−catenin *(arrows).*

Immunoreactivity for FZC18, β−catenin and for the positive target of Wnt signaling glutamine synthetase (GS) was assessed on serial sections of normal, cirrhotic livers and HCCs (Figure 7). The intensity was semi-quantitatively recorded using a 5-point scale, from absent (−) to strong (++++), by comparing the staining in the tumor with adjacent non-tumor tissue, as described [26]. In normal liver, FZC18 was periportal (Figure 7A), contrasting with the well-characterized pericentral vein localization of GS (Figure 7B) [13], indicating that FZC18 epitopes are detected in zones of low β−catenin pathway activation.

In HCCs, mild FZC18 signal was detected at sites where GS was strong and β−catenin was cytoplasmic or nuclear (Figure 7, D–F). Conversely, strong FZC18 signal was detected at sites where GS staining was mild and β−catenin was associated with cell membranes (Figure 7, G–L). Consistently, statistical analysis of data from 24 tumor nodules indicated that FZC18 was negatively correlated with GS (γ = −0.42; p = 0.02; n = 24) and cytoplasmic β−catenin staining (γ = −0.47; p = 0.02; n = 23). Conversely, FZC18 was positively correlated with cell membrane β−catenin staining (γ = 0.67; p<0.001; n = 23). As expected [26], nuclear and cytoplasmic β−catenin were positively correlated (γ = 0.89; p<0.001; n = 23), as well as GS and nuclear (γ = 0.74; p<0.001; n = 23) or cytoplasmic β−catenin (γ = 0.63; p<0.001; n = 23). These data suggest that the FZC18 module could inhibit Wnt/β−catenin signaling in the tumor microenvironment.

### Proteolytic processing of V3C18 in cancer tissues *in vivo*

**Figure 8:**
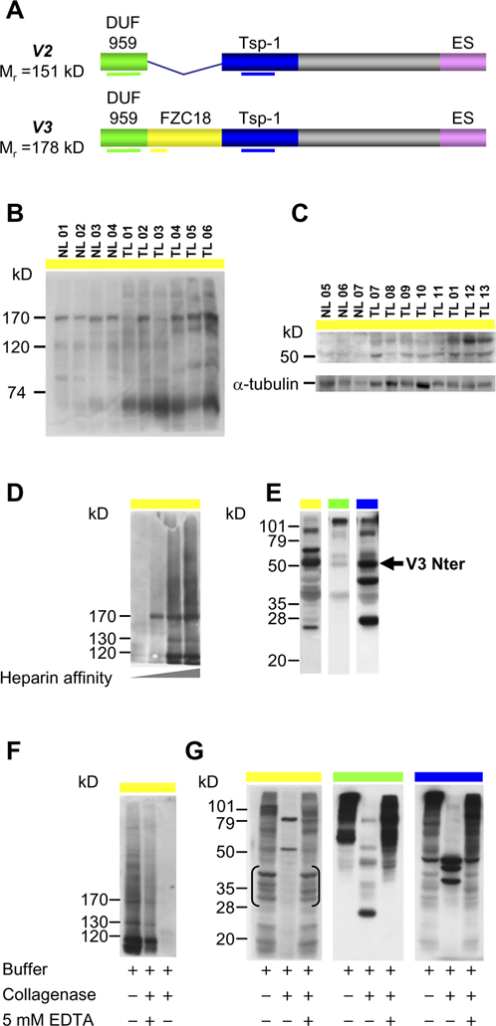
*In vivo* processing of the N-terminus of V3C18 releases a 50 kD FZC18 precursor. (A) Schematic of V2 and V3 of C18 showing DUF-959, FZC18, Tsp-1C18 (thrombospondin-1) and ES (endostatin) modules. Thick horizontal color lines indicate the antibodies used. (B and C) Histologically representative 5-µm freeze-dried cryosections from human normal (NL) and tumor livers (TL) were scraped off slides with sterile scalpels, solubilized in 10% SDS buffer and dosed. Two hundred µg of whole protein extract was resolved by denaturing 5% (B) or 12% (C) PAGE-SDS, followed by immunoblot. NLs show a distinct ∼170 kD polypeptide (B). TLs show variable levels of the ∼170 kD polypeptide, a prominent polypeptide band below the 74 kD marker (B) and ∼50 to 60 kD bands (C). After membrane stripping, mouse monoclonal anti-α-tubulin antibody is used as a loading control. (D–G) A frozen human HCC tissue block (case TL 06, shown in B) was homogenized using a tissue grinder in non-denaturing buffer containing protease inhibitors at +4°C. Proteins were clarified by ultracentrifugation (130 000 *g),* fractionated by heparin-affinity chromatography and analyzed by denaturing 5% (D and F) or 12% (E and G) PAGE-SDS and immunoblot. (D) 170-kD V3C18 binds heparin with high affinity. (E) Non heparin-binding polypeptides from the N-terminus of C18: a ∼50 kD polypeptide is detected by the three antibodies *(V3Nter, arrow)*. (F and G) Digestion of heparin-binding proteins with bacterial collagenase releases N-terminal noncollagenous polypeptides containing FZC18 and DUF-959 epitopes. Collagenase activity is inhibited by EDTA, suggesting that V3Nter can be released by a metalloprotease. Three identical sample sets were gel-migrated and blotted in parallel, then cut into 3 stripes before immunodetections. [Anti-FZC18 detects nonspecific polypeptides between 30–40 kD *(brackets)*].

Immunoblot analysis using anti-FZC18 antibody on whole protein extracts from histologically representative cryosections from normal and tumor livers (Figure 8, B and C) showed a ∼170 kD polypeptide consistent with V3FL (M_r_ = 178 kD) in 4/4 normal and 4/6 tumor livers (Figure 8B). In addition, a prominent polypeptide band was detected below the 74 kD marker in all tumors but not in normal livers (Figure 8B). Search for further proteolyzed forms in additional normal and tumor samples showed two polypeptides above the 50 kD marker in 7/7 tumors (Figure 8C). The tumor sample 01 (TL 01) was included both in Figure 8B and 8C, showing predominantly proteolyzed forms. These data show a higher ratio of proteolyzed over full-length forms in cancerous tissues and a higher ratio of full-length over proteolyzed forms in normal tissues. Thus, an important fraction of FZC18 detected by immunohistochemistry in HCCs corresponds to processed C18 polypeptides containing the *frizzled* CRD module, implying that the negative correlation between FZC18 staining and Wnt/β−catenin activity in cancer tissues (see above) substantially corresponds to processed V3C18.

Protein homogenates from the HCC case TL 06 containing both full-length and proteolyzed forms (Figure 8B) were fractionated by heparin-affinity chromatography, because C18 binds heparin through C-terminal endostatin [23]. Heparin-binding full-length (∼170 kD) and partially processed FZC18-containing polypeptides (∼130 and ∼120 kD) (Figure 8D) were separated from non heparin-binding ones (Figure 8E). Among non heparin-binding proteins, a 50 kD polypeptide contained the FZC18, Tsp-1C18 and DUF-959 epitopes (Figure 8E). Digestion of endogenous V3C18 with collagenase (Figure 8F) released 80 kD and 50 kD polypeptides, the reaction being inhibited by EDTA (Figure 8G), indicating that these polypeptides can be released by a metalloprotease. Interestingly, a ∼26 kD polypeptide was detected by anti-DUF-959 only (Figure 8G). Its molecular weight is consistent with DUF-959 (M_r_ = 20 kD). In addition, the anti-Tsp-1C18 antibody detected three collagenase-resistant polypeptides (48; 44 and 32 kD), but not the 50 kD polypeptide (Figure 8G). Thus, the 50 kD polypeptide released *in vitro* by collagenase digestion of endogenous C18 is a partially processed form of V3C18 containing the FZC18 and DUF-959 modules, which confirms the existence of V3Nter *in vivo*.

We previously showed that full-length V2C18 is an abundant plasma protein secreted by the liver in healthy human subjects [9]. Consistently, unprocessed V3C18 was detected in normal liver (Figure 8B), suggesting that the periportal signal seen by immunohistochemistry (Figures 7A and S4A) corresponds mainly to V3FL. Since V3FL is predominantly detected in liver zones characterized by low Wnt/β−catenin activity levels (Figure 7, A–C), it would be tempting to speculate that the expression of V3C18 could be negatively regulated by Wnt/β−catenin signaling. However, we also showed that some of the FZC18 epitopes may be masked by sialic acid (Figure 4E). Therefore, antibody-based analysis of V3C18 does not necessarily reflect expression, as detection can be influenced by post-translational processing.

### Differential topology of FZC18 and DUF-959 *in vivo*

Consistently with pericellular matrix localization of FZC18 *in vitro,* FZC18 highlighted intercellular spaces in human primary liver cancer tissues (Figure S4B) and was detected either at the surface of tumor cells or as supranuclear intracellular material (Figure S4D), as shown by a tridimensional reconstruction of FZC18 at the cell surface in human liver cancer case TL 03 (Movie S2). In this case, cell surface signal likely corresponds to fully proteolyzed FZC18-containing V3C18 polypeptides, as was shown in Figure 8B.

V3C18 (Figure S4D) and V2C18 (Figure S4E) showed a highly contrasting topology in contiguous tissue sections from liver cancer, V3C18 highlighting individual cells and V2C18 outlining tumor sinusoids. Anti-DUF-959 antibody detects both V3Nter and V3FL, as well as both V2Nter and V2FL (see *eg.,* Figures 4A and 8A), whereas anti-FZC18 detects FZC18, V3Nter and V3FL. Bearing this in mind, the highly different signal obtained with anti-FZC18 (Figure S4D) and anti-DUF-959 (Figure S4E) antibodies in contiguous sections of liver cancer was surprising. Several lines of evidence explain these data.

First, using the normal human total mRNA master panel shown in Figure 1B, we compared the mRNA expression levels of V2C18 and V3C18 by QRT-PCR. The expression of V3C18 was several folds lower than that of V2C18 mRNAs. Mean ΔCT were 12.2±1.6 for V2C18 and 16.1±1.3 for V3C18 (p = 4.5^−8^, Mann-Whitney's U Test), consistently with previous data showing that V3 is expressed at very low levels [3], [7], [27].

Second, we confirmed these data by probing a panel of 8 normal, 12 cancer and 7 inflammatory liver tissues by Northern blot with ^32^P-labeled cDNA probes, revealing film exposure times several folds longer for V3C18 than for V2C18 to detect signal (data not shown). Thus, given the much higher expression levels of V2C18 than of V3C18 in all tissues [3], [7], [27], immunohistochemical detection of endogenous C18 with anti-DUF-959 antibody reveals the quantitatively predominating V2C18 signal, whereas anti-FZC18 antibody detects only V3C18. Consistently, comparison of Figure S4, D and E, shows higher signal intensity with anti-DUF-959 antibody. In addition, as shown in Figure 4E, antibody-based analysis of V3C18 strictly reveals unmasked FZC18 epitopes. As unmasking of FZC18 can be achieved by clipping off the sialylated DUF-959 module (Figures 4 and S2) and immunostaining in tumors reveals mainly proteolytically processed FZC18-containing polypeptides (Figure 8), the striking differences between Figures S4, D and E denote differential topology of FZC18 and DUF-959 modules, respectively.

## Discussion

We identified FZC18 as a novel extracellular inhibitor of Wnt/β−catenin signaling. FZC18 decreases tumor cell growth through increased cell death in human cancer cell lines carrying activating β−catenin mutations. In human liver cancers, C18 is processed to *frizzled* CRD-containing polypeptides and FZC18 immune reactivity negatively correlates with active Wnt signaling. Moreover, FZC18 localizes at the cell surface and pulls down Wnt3a *in vitro*, suppressing Wnt-dependent activation of β−catenin signaling. Furthermore, proteolytic processing regulates the biological activity of FZC18, as full-length C18 does not suppress β−catenin regulated gene expression.

### FZC18 inhibits Wnt signaling in cancer cells carrying activating β−catenin mutations

We show that exogenous wild-type β−catenin increases CRT, *CCND1* promoter activity and tumor cell survival in HCT116 and in HepG2 cells carrying heterozygous β−catenin ΔS45 and Δ25-140, respectively, thus lacking key phosphorylable residues in one of the alleles. Recent reports showed that HCT116 cells express canonical Wnts [28] and respond to exogenous Wnt1 by increasing CRT [15]. Thus, oncogenic β−catenin mutations do not saturate TCF-dependent transcription in cancer cells. Consistently, in HCT116 and HepG2 cells, SFRP1, SFRP5 and FZC18 decrease CRT, *CCND1* promoter activity and cell survival and thereby induce cell death. In agreement with our data, overexpression of SFRP1, SFRP2 and SFRP5 in HCT116 and SW480 CCR cells destabilize ectopically expressed β−catenin ΔS45 [15]. Taken together, these data indicate that FZC18 destabilizes β−catenin in cells carrying heterozygous β−catenin activating mutations.

### FZC18 reduces tumor cell growth

In CRC and HCC cell lines, FZC18 decreases the protein levels of total and non-phosphorylated β−catenin and downstream targets, such as cyclin D1 and *c-myc.* These findings are associated with ∼60% to 70% reduction of tumor cell growth and ∼25% induction of cell death. These data are consistent with the fact that SFRP1, SFRP2 and SFRP5 suppress Wnt-dependent transcription by 60%, decrease *c-myc* mRNA expression, inhibit *in vitro* tumor growth and induce cell death in CRC cells carrying oncogenic mutations of β−catenin [15]. Similarly, SFRP1 sensitizes breast cancer cells to proapoptotic stimuli [29] and SFRP3 suppresses tumor growth and cell invasion in prostate cancer cells [30].

Wnt-induced β−catenin stabilization is an important survival signal which regulates homeostasis [31] and increases the threshold for contact inhibition and anchorage-independent cell growth [32]. As a result, reduction in tumor cell growth can be achieved by either extracellular quenching of Wnts with monoclonal antibodies [33], [34], β−catenin knockdown [35] or restoring normal APC [36].

FZC18 turns off *c-myc* in CRC cells, which may explain the induction of cell death. Indeed, Wnt signaling disables the checkpoint of *c-myc*-induced apoptosis in tumor cells [37], thus enhancing its prooncogenic effects [38]. Although *c-myc* is a target of β−catenin in CRCs [39], its link with β−catenin in HCCs remains controversial [40], [41]. In addition, FZC18 turns off cyclin D1 in CRC and HCC cancer cell lines. Cyclin D1 is a downstream effector of β−catenin, promoting S-phase entry and tumor growth [42] and is frequently upregulated in HCCs [41]. The common theme in these approaches is the inhibition of the Wnt/β−catenin pathway at different levels, providing a rationale for therapeutic intervention [36]. Particularly, inhibiting Wnt-FZ interaction at the cell surface was recently indicated as a viable strategy for anti-cancer treatment [43].

### Ectopic FZC18 binds Wnt3a *in vitro*

We show that ectopically expressed Wnt3a pulls down FZC18 *in vitro* and that FZC18 suppresses Wnt3a-dependent activation of Wnt signaling in human cancer and embryonic kidney cells. Additionally, homology modeling shows 100% probability that the FZC18 CRD structure matches FZ8 and SFRP3 CRDs. Moreover, a peptide lying at the solvent-exposed surface of FZC18 CRD competes with the FZC18-Wnt3a interaction. This peptide partially overlaps residues previously shown to be essential to Wnt-FZ8 interactions [25]. Taken together, these data suggest that both partners could be associated at the cell surface and favor the hypothesis of Wnt quenching by FZC18. Alternatively, the interaction of FZC18 with the cell surface could occur *via* heterodimerization with FZ receptors, as has been shown for SFRPs [44] or through as yet unidentified receptors.

### FZC18 is negatively associated with Wnt/β−catenin pathway activity *in vivo*

In liver cancers, FZC18 immunostaining negatively correlates with Wnt/β−catenin signaling and with the protein expression of the β−catenin-regulated gene GS, consistently with the suppression of GS synthesis by DKK1 in mice carrying an APC disruption [13]. Immunohistochemistry with the anti-FZC18 antibody cannot distinguish between V3FL and processed V3C18 polypeptides containing the *frizzled* CRD. However, immunoblot analysis showed that V3C18 is predominantly processed in liver cancers, yielding the FZC18-containing polypeptide V3Nter, which inhibits Wnt/β−catenin signaling. In addition, small, well-differentiated liver cancers express higher levels of V3C18 mRNA than large, less differentiated ones. Taken together, these results suggest that FZC18 can downregulate β−catenin activation and expression of downstream genes, such as GS, a marker of activating β−catenin mutations in HCCs [26]. Consistently, in chemically-induced carcinogenesis in rodents, GS (+) HCC foci grow faster than GS (−) ones and the GS (+) phenotype is associated with larger tumors, β−catenin mutations and decreased apoptosis [45]. As glutamine is critical for the growth of highly proliferating cells, supporting protein and nucleotide synthesis and providing a major source of energy [46], GS could make the cell independent of glutamine supply, conferring a growth advantage.

### Bioactive FZC18 is encrypted within and released from V3C18

The N-terminus of V3C18 undergoes stepwise proteolytic processing, involving release of a 50 kD biologically active precursor consisting of DUF-959, FZC18 and part of Tsp-1C18, that we called *V3Nter.* Release of V3Nter from endogenous V3C18 was inhibited by EDTA, suggesting that a metalloprotease (MMP) may be involved in decrypting FZC18 biological activity. MMPs are major players of ECM proteolysis at the cell surface in tumor invasion and metastasis, exposing and activating cryptic epitopes from, *e.g.,* laminins, enhancing cell migration or from plasminogen, inhibiting angiogenesis. Thus, MMPs precisely control cell function and are therefore viewed as signaling molecules [47].

V3Nter is N-glycosylated, consistently with previous bioinformatics predictions [3]. Remarkably, sialylation masks some of the epitopes detected by the anti-FZC18 antibody because the ∼50; ∼48 and 36 kD polypeptides are only detected after Sialidase A digestion. Excision of DUF-959 from this precursor releases a FZC18-containing 36 kD N-glycosylated polypeptide. Wnts and SFRPs are N-glycosylated [21]. Glycosylation may be involved in apical cell-surface sorting and secretion of Wnts bound to lipoprotein particles, thus facilitating long-range signaling [48]. In addition, both N-glycosylation and sialylation may participate with disulfide bonding in dimerization [49]. Moreover, DUF-959 contains a conserved coiled-coil motif [7], which may be involved in oligomerization. Thus, glycans and conserved motifs may regulate the release of soluble and active FZC18.

In summary, we show that C18 contains a functional *frizzled* CRD that we named FZC18. The biological activity of FZC18 is cryptic because full-length *frizzled* CRD-containing C18 is unable to inhibit Wnt signaling. Rather, full-length C18 may activate Wnt signaling regardless of the FZC18 module, as V2FL potently induces CRT. Human V1C18 is a HSPG and its HS attachment sites are not variant-specific [8]. Consistently, we show that V3C18 is a cell surface HSPG *in vivo* and *in vitro*. HS enhance Wnt signaling by raising local concentrations of Wnts at the cell surface, possibly promoting Wnt interaction with the FZ-LRP receptor complex [21]. Taken together, these data show for the first time a collagen containing a cryptic *frizzled* CRD, which can be released and activated by proteolysis. Moreover, the expression of *frizzled* CRD-containing C18 increases during fibrogenesis and in small, well-differentiated cancers with a favorable outcome.

## Materials and Methods

### Cell culture, tissue samples and mRNA expression analysis

Human CRC cell lines HCT116, HT29 and Caco-2 were cultured in McCoy's 5A plus 10% FCS (Invitrogen). Human HCC cell lines HepG2, Huh-6, Huh-7 [20], HepaRG [50], BC1, BC2 and B16A2 [51] and the mouse HCC cell lines mhAT3F and mhAT3FS315 [18], were cultured as described. MCF-7, HEK293T and 293EBNA cells were cultured in DMEM (Invitrogen), plus 10% FCS. Human tissue samples and mRNA were obtained as described [10], complying with the guidelines of the National Steering Committee of HCC (INSERM, Paris). Relative mRNA expression was assessed using mRNA arrays hybridized with ^32^P-labelled cDNAs normalized to 18S under linear-range conditions [10] or by QRT-PCR using SYBR Green PCR Master Mix (Applied Biosystems) and the ABI Prism 7000 (Perkin Elmer). Expression was normalized to 18S and to a calibrator. Primers were designed with Primer 3 on the www. Sequences and conditions are available on request.

### cDNA clones

Full-length V2 and V3 C18 cDNAs were described [7]. Human V2Nter and V3Nter cDNAs were PCR-cloned in frame with a V5 tag into pcDNA3.1. Mouse V2Nter and V3Nter were cloned into pREP7 (Invitrogen). Human FZC18 was PCR-cloned in pCRII (Invitrogen) using V3Nter as a template, excised with EcoRI and cloned into pSecTag2 (Invitrogen) in frame with an IgΚ signal sequence and a C-terminal *myc* tag. Mouse Wnt1 (pV101, from R. Nusse) and Wnt3a (in pBSII KS+, from J. Kitajewski) cDNAs were transferred to pcDNA 3.1 (Invitrogen) in frame with a C-terminal poly-His tag. SFRP1 and 5 in pcDNA3.1/HisC were from S. Baylin [15]. Super8XTOP- and Super8XFOP-FLASH reporters were from R. Moon [52]. The Cyclin D1 promoter reporter D1Δ-944pXP2 was from J. Pouyssegur [22]. The normalization Renilla luciferase vector pGL4.70[*hRluc*] was from Promega. Wild-type β−catenin cDNA was from R. Grosschedl [53]. All cDNAs were checked by automatic sequencing (Sequencing Facility, Rennes Hospital, France).

### Reporter assays

Cells (5×10^4^/well) were transfected on 24-well plates with Lipofectamine Plus (Invitrogen). cDNA was normalized to 250 ng with the appropriate empty vectors. Super8XTOP/FOP Flash reporters (15 ng each) or Cyclin D1 promoter reporter (100 ng) were cotransfected with pGL4.70[*hRluc*] expressing Renilla luciferase. After 48 h, luciferase activity was measured in a scintillation counter (LS6500, Beckman) using the Dual Luciferase Reporter Assay System (Promega).

### Antibodies and immunological methods

Anti-C18 antibodies detected human DUF-959, Tsp-1C18 [8], FZC18 [7] and endostatin [54] as described and mouse DUF-959 (Saarela et al., unpublished). Monoclonal mouse antibodies were directed against: Hsc70, *c-myc* (9E10) and β−catenin (E-5) (Santa Cruz); non phosphorylated β−catenin (8E4 Upstate), *myc* and V5 tags (Invitrogen), penta-His tag (Qiagen) and glutamine synthetase (BD Biosciences). Polyclonal rabbit anti-cyclin D1 was from Labvision. Secondary antibodies were sheep anti-mouse or goat anti-rabbit coupled to peroxidase (Biorad) or sheep anti-mouse and goat anti-rabbit coupled to FITC or TRITC (Sigma), respectively. Immunoblots and immunohistochemistry were done as described [9]. Blot image files were processed with MultiGauge (FujiFilm Lifescience). Microscopes used: Olympus BX60 or confocal Leica TCS NT system on a Leica DMB microscope. Color digital files were prepared with Adobe RGB (1998) on Adobe Photoshop 7.

For immunoprecipitation, mouse His-tagged Wnt3a and mouse V3Nter or V2Nter cDNAs were cotransfected in HEK 293-EBNA cells. Cell lysates were incubated with sheep-anti-mouse-IgG-coated Dynabeads M-280 (Dynal) conjugated with anti-His. After washing in RIPA buffer, complexes were eluted in denaturing sample buffer, resolved by 10% PAGE-SDS and immunoblotted with anti-His antibody (to detect Wnt3a) or with anti-DUF-959 (to detect V3Nter or V2Nter). To compete with the Wnt3a-V3Nter interaction, the synthetic peptide RH3 (AWGRFLHTNCHPFLA) from V3Nter CRD was added to culture media.

### Posttranslational modifications of C18

Proteoglycans were purified as described [8]. Briefly, 900 mg from a frozen tumor was homogenized in 4M guanidine buffer, clarified by centrifugation and equilibrated in 8M urea buffer. Proteoglycans were isolated by batch anion-exchange on Q-Sepharose (GE Healthcare), followed by HiTrap-Q and eluted with NaCl (0.5M–1.5M). One µg of proteoglycan was digested in 4 mIU of heparin lyase II or III (Sigma), 120 mU of Chondroitinase ABC, (Seikagaku, Japan) or 20 U of collagenase (Worthington, USA). Proteins from mhAT3FS315 cells expressing V2Nter or V3Nter cDNAs were digested with N-glycosidase and Sialidase A (Glyko Enzymatic Deglycosylation kit, Prozyme). Heparin-binding proteins were isolated from 500 mg of frozen human HCC homogenized in non denaturing buffer with protease inhibitors (Sigma), clarified by ultracentrifugation and applied to a 5-ml HiTrap heparin column. After extensive washing, bound proteins were eluted stepwise (0.05−2M NaCl) [23].

### Colony formation assay and flow cytometry

Assays were performed as described [15]. Cells were transfected using Lipofectamine Plus (Invitrogen), stripped and plated in triplicates in 100-mm (colony formation) or 6-well plates (flow cytometry) 24 h after transfection and selected with 0.6 mg ml^−1^ G148 or 0.5 mg ml^−1^ zeocin (Invitrogen).

### Statistics

Differences between means were assessed by the Mann-Whitney's “U” test. Bivariate relationships were calculated by the Spearman's rank-order correlation coefficient *R* or Goodman-Kruskal's *Gamma* (Statistica 7.1, StatSoft 2007).

## Supporting Information

Figure S1 V3FL localizes at the cell surface, but does not inhibit Wnt/ β-catenin signaling. (A) Schematic of V2FL and V3FL showing DUF-959, FZC18, Tsp-1C18 (thrombospondin-1) and ES (endostatin) modules. Thick horizontal lines indicate the antibodies used. (B–D) Overlays of immunofluorescence and phase contrast confocal microscopy of mhAT3FS315 hepatoma cells transiently transfected with V3FL (B) or V2FL (C and D) cDNAs and probed with anti-Tsp-1C18, followed by anti-mouse FITC-labeled IgG (*green*). V3FL highlights the cell membranes (B, *arrows*). V2FL is detected within the cell (C); by contrast, cell membranes and intercellular boundaries show no signal (*arrows*). V2FL (D) is detected within a large vacuole (*v*) indented by the nucleus (*N*) resembling a Golgi structure. The cell surface shows a secretion vesicle (*arrow*) and fraying-like material (*arrowhead*) suggesting secretion of V2. (E) Changes in CRT in response to increasing amounts of transiently transfected cDNA vectors. Reporter gene assays using a β-catenin-TCF reporter driven by wild-type (*SUPER8XTOPFLASH*, white bars) or a negative control with mutated TCF binding sites (*SUPER8XFOPFLASH*, black bars). Results are means of three replicates from a representative experiment. Three independent experiments were performed. Error bars represent standard deviations. (F) Reporter gene assays using a β-catenin-TCF responsive reporter (*SUPER8XTOPFLASH*) in human HCC Huh-7 cells (wild-type β-catenin). Results are means of three replicates from a representative experiment. Three independent experiments were performed. Error bars represent standard deviations. *P* = (Student's “*t*” test) indicates statistical significance with respect to cells transfected with vector alone (*VECTOR*). *NS*, not significant. (G) *Left*: Protein extracts from a human HCC (case TL 06, see Figure 8, B, D–G and Figure S4, D and E) were chromatographed through Q-Sepharose and eluted with 0.5M (lane 1) and 1M (lane 2) NaCl. *Right*: One µg from the 1M NaCl fraction was incubated with the indicated enzymes. Protein samples were resolved by denaturing 5% PAGE-SDS and immunoblotted. Heparinase digestion results in mobility shift and epitope unmasking. (H) V2FL and V3FL cDNAs were transiently transfected in mhAT3FS315 mouse hepatoma cells. Protein samples from cell layers and from conditioned media were resolved by denaturing 5% PAGE-SDS and immunoblotted. Both V2FL and V3FL show a smear-like migration profile typical of proteoglycans. V2FL is soluble in conditioned medium. V3FL is detected at high levels in cell layers but is not detected in conditioned medium. (I) Reporter gene assays using cyclin D1 promoter reporter upstream luciferase cDNA in HCT116 and HepG2 cells. Results are means of three replicates from a representative experiment. Three independent experiments were performed. Error bars represent standard deviations. *P* = (Student's “*t*” test) indicates statistical significance with respect to cells transfected with vector alone (*VECTOR*). *NS*, not significant.
(4.22 MB TIF)

Figure S2 Sialylation of V3Nter resides within the DUF-959 module. (A) Schematic of V2Nter (*left*) and FZC18 (*right*) cDNA vectors and antibodies. Horizontal color lines below each module denote the antibodies used. (B) Immunoblot of mhAT3FS315 cell conditioned medium after transfection with FZC18 cDNA. Proteins were separated in denaturing 7.5% PAGE-SDS. The asterisk (*) denotes immunoglobulins from FCS-containing medium. N-glycosidase digestion induces a ∼3kD mobility shift of the FZC18 module detected by anti-FZC18 and by anti-*myc* tag antibodies. (C) N-glycosidase and Sialidase A digestion of V2Nter. Protein extracts from cell layers were incubated at 37 {degree sign}C for 2 hr with N-glycosidase and Sialidase A or with buffer alone. Sialidase A reveals polypeptides of ∼30 kD and 32 kD.
(0.76 MB TIF)

Figure S3 A 15-amino acid peptide derived from the CRD of FZC18 (RH3 peptide) competes with FZC18 binding to Wnt3a. (A) EBNA-293 cells were cotransfected with mouse V3Nter and with His-tagged mouse Wnt3a. Transfected cells were incubated with 0; 50 or 100 µg×ml^−1^ of the synthetic peptide RH3 from the CRD domain of FZC18. Cell lysates were analyzed by immunoblot (10% PAGE-SDS) or coimmunoprecipitated (*IP*) with monoclonal anti-His antibody. (B) 3D structure prediction of the FZC18 CRD and modeling of the potential surfaces involved in Wnt-FZC18 interactions. SFRP3 and Frizzled-8 CRD crystal structures were used as templates. The orientation of the CRD surface on the right is rotated 180° about the vertical axis with respect to left-side images. Blue, N-termini; gray, C-termini; green, surfaces involved in Wnt-CRD interactions inferred from structure-based alignment of FZC18, SFRP3 and FZ8 CRDs and from described mutations affecting Wnt-CRD binding [25]. Red, localization of the RH3 peptide. Yellow, red and green overlay. 3D structure prediction was done using the *Phyre* www server and Protein Explorer 2.79.
(1.74 MB TIF)

Figure S4 Detection of FZC18 in human liver. (A) Immunoperoxidase staining (*brown*) in normal human liver shows FZC18 in periportal hepatocytes. In the portal tract (*PT*), including portal veins (*v*), bile ducts and basement membrane (*arrow*) no signal is detected. Case NL 04, shown in Figure 8B. (B) HCC. Speckle-like FZC18 (*arrows*) is seen on the surface of tumor cells highlighting intercellular spaces (case TL 03, shown in Figure 8B). (C) Liver cirrhosis shows speckle-like FZC18 in hepatocytes (*H*). No FZC18 is seen in the surrounding stroma (*S*). (D and E) Case TL 06, shown in Figure 8, B and D–G. Contrasting topology of FZC18 (D) and DUF-959 (E) in HCC. FZC18 highlights the pericellular matrix (*arrows*) and is also detected as supranuclear intracellular material (*arrowheads*). In the same tumor, DUF-959 outlines cords of tumor cells highlighting tumor sinusoids (*s*).
(9.05 MB TIF)

Movie S1 3D cell surface reconstruction of V3Nter *in vitro*. Confocal fluorescence microscopy of mhAT3FS315 mouse liver cancer cells transiently transfected with V3Nter cDNA and probed with anti-DUF-959, followed by anti-rabbit TRITC-labeled IgG (*red*). Original magnification×1000. Fifteen representative images acquired at 500-nm intervals on the vertical axis were stacked on ImageJ 1.37v (NIH, USA).
(0.13 MB MOV)

Movie S2 3D cell surface reconstruction of FZC18 *in vivo*. Reconstruction of FZC18 staining at the cell surface in a human hepatocellular carcinoma (case TL 03, shown in Figures 8B and S4B). Original magnification×1000. Images were acquired on the vertical axis and stacked on ImageJ 1.37v (NIH, USA). In the light of data on Figure 8B, cell surface signal shows proteolyzed FZC18-containing V3C18 polypeptides.
(0.13 MB MOV)
