# Supplementary figures and images for: A Cryptic Frizzled Module in Cell Surface Collagen 18 Inhibits Wnt/β−Catenin Signaling
Source: PLoS One. 2008 Apr 2;3(4):e1878. doi: 10.1371/journal.pone.0001878 (PMC2270346; doi:10.1371/journal.pone.0001878)

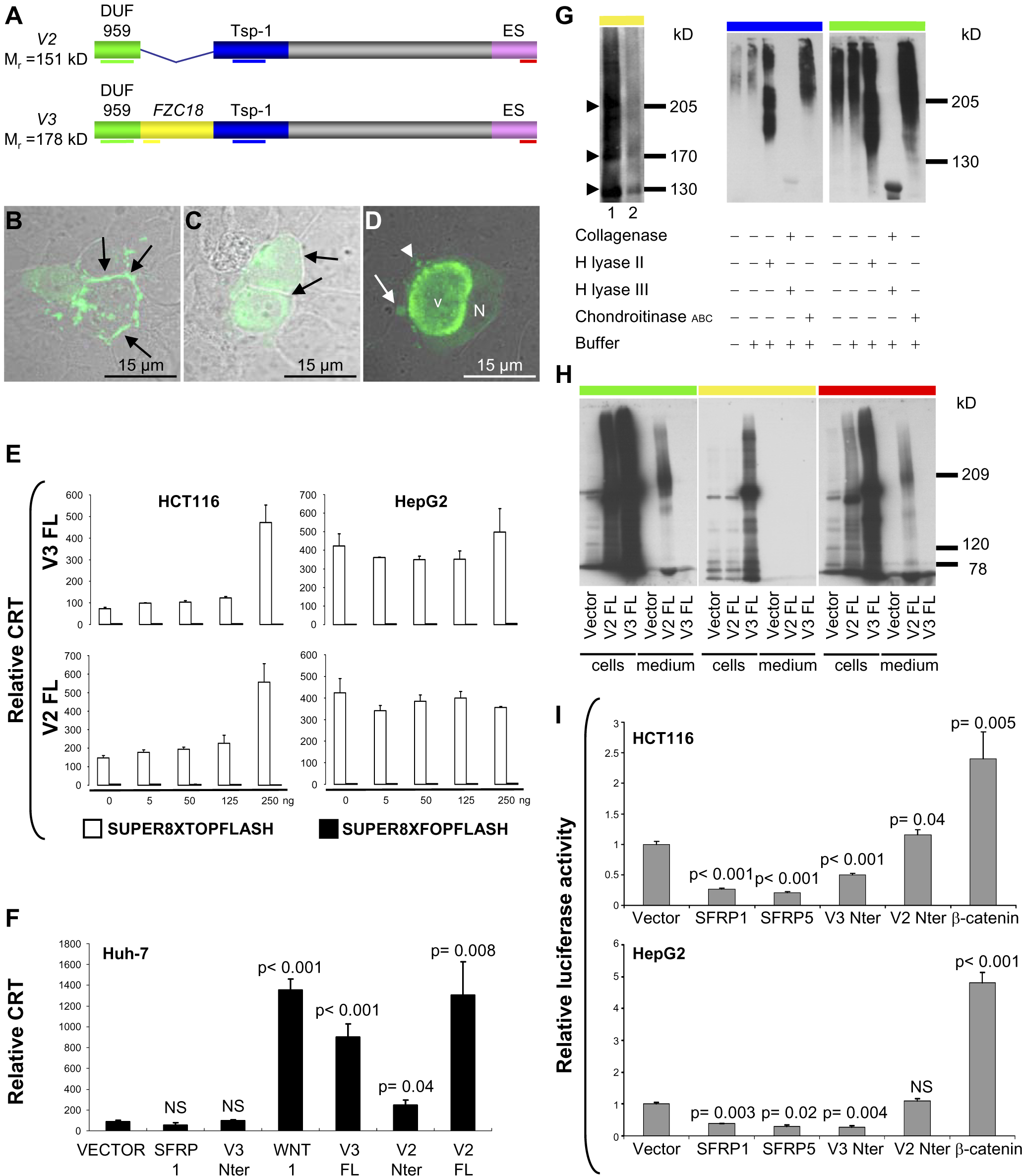

Supplement: Figure S1 — V3FL localizes at the cell surface, but does not inhibit Wnt/ β-catenin signaling. (A) Schematic of V2FL and V3FL showing DUF-959, FZC18, Tsp-1C18 (thrombospondin-1) and ES (endostatin) modules. Thick horizontal lines indicate the antibodies used. (B–D) Overlays of immunofluorescence and phase contrast confocal microscopy of mhAT3FS315 hepatoma cells transiently transfected with V3FL (B) or V2FL (C and D) cDNAs and probed with anti-Tsp-1C18, followed by anti-mouse FITC-labeled IgG (green). V3FL highlights the cell membranes (B, arrows). V2FL is detected within the cell (C); by contrast, cell membranes and intercellular boundaries show no signal (arrows). V2FL (D) is detected within a large vacuole (v) indented by the nucleus (N) resembling a Golgi structure. The cell surface shows a secretion vesicle (arrow) and fraying-like material (arrowhead) suggesting secretion of V2. (E) Changes in CRT in response to increasing amounts of transiently transfected cDNA vectors. Reporter gene assays using a β-catenin-TCF reporter driven by wild-type (SUPER8XTOPFLASH, white bars) or a negative control with mutated TCF binding sites (SUPER8XFOPFLASH, black bars). Results are means of three replicates from a representative experiment. Three independent experiments were performed. Error bars represent standard deviations. (F) Reporter gene assays using a β-catenin-TCF responsive reporter (SUPER8XTOPFLASH) in human HCC Huh-7 cells (wild-type β-catenin). Results are means of three replicates from a representative experiment. Three independent experiments were performed. Error bars represent standard deviations. P = (Student's “t” test) indicates statistical significance with respect to cells transfected with vector alone (VECTOR). NS, not significant. (G) Left: Protein extracts from a human HCC (case TL 06, see Figure 8, B, D–G and Figure S4, D and E) were chromatographed through Q-Sepharose and eluted with 0.5M (lane 1) and 1M (lane 2) NaCl. Right: One µg from the 1M NaCl fraction was [file pone.0001878.s001.tif]

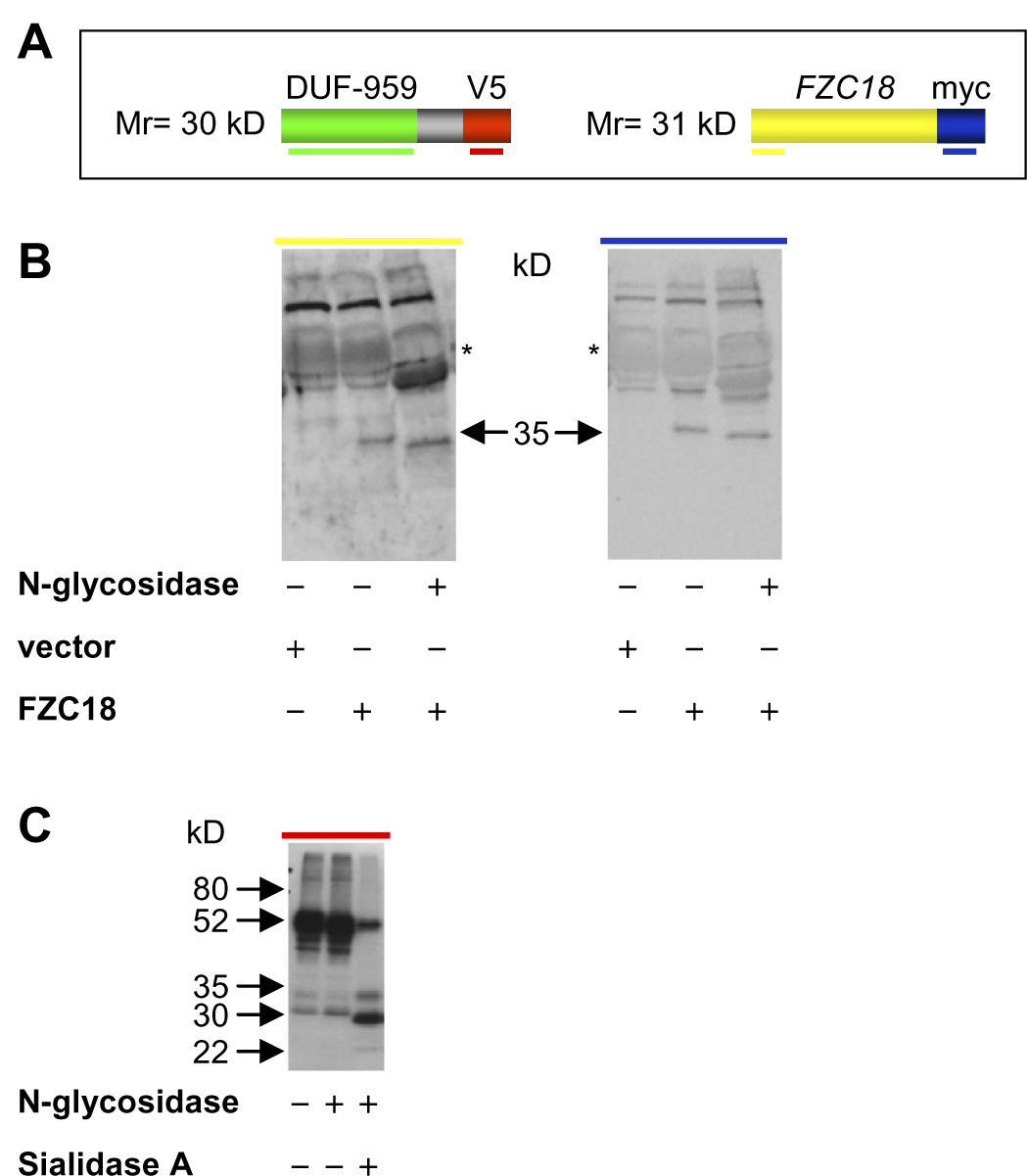

Supplement: Figure S2 — Sialylation of V3Nter resides within the DUF-959 module. (A) Schematic of V2Nter (left) and FZC18 (right) cDNA vectors and antibodies. Horizontal color lines below each module denote the antibodies used. (B) Immunoblot of mhAT3FS315 cell conditioned medium after transfection with FZC18 cDNA. Proteins were separated in denaturing 7.5% PAGE-SDS. The asterisk (*) denotes immunoglobulins from FCS-containing medium. N-glycosidase digestion induces a ∼3kD mobility shift of the FZC18 module detected by anti-FZC18 and by anti-myc tag antibodies. (C) N-glycosidase and Sialidase A digestion of V2Nter. Protein extracts from cell layers were incubated at 37 {degree sign}C for 2 hr with N-glycosidase and Sialidase A or with buffer alone. Sialidase A reveals polypeptides of ∼30 kD and 32 kD. (0.76 MB TIF) [file pone.0001878.s002.tif]

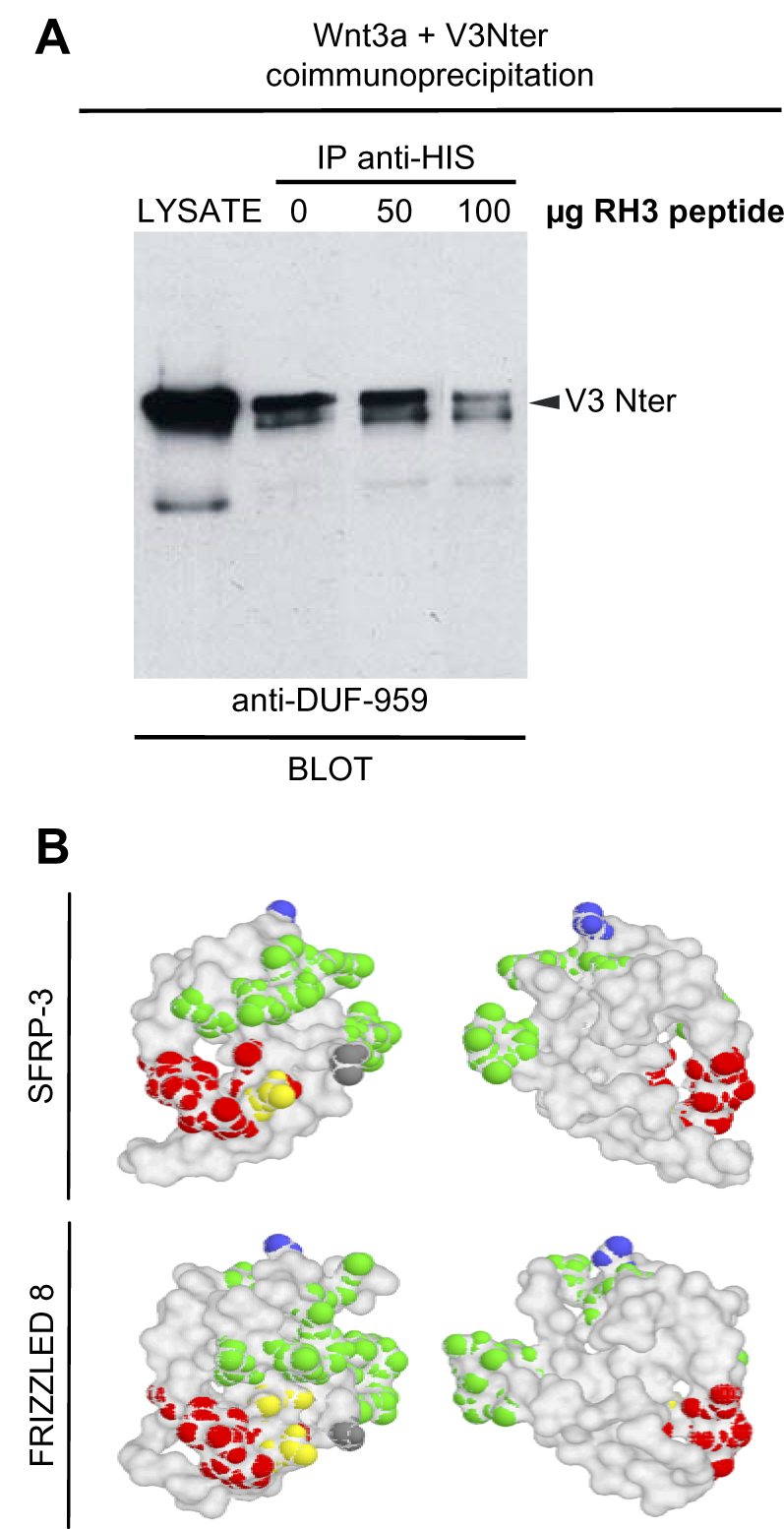

Supplement: Figure S3 — A 15-amino acid peptide derived from the CRD of FZC18 (RH3 peptide) competes with FZC18 binding to Wnt3a. (A) EBNA-293 cells were cotransfected with mouse V3Nter and with His-tagged mouse Wnt3a. Transfected cells were incubated with 0; 50 or 100 µg×ml−1 of the synthetic peptide RH3 from the CRD domain of FZC18. Cell lysates were analyzed by immunoblot (10% PAGE-SDS) or coimmunoprecipitated (IP) with monoclonal anti-His antibody. (B) 3D structure prediction of the FZC18 CRD and modeling of the potential surfaces involved in Wnt-FZC18 interactions. SFRP3 and Frizzled-8 CRD crystal structures were used as templates. The orientation of the CRD surface on the right is rotated 180° about the vertical axis with respect to left-side images. Blue, N-termini; gray, C-termini; green, surfaces involved in Wnt-CRD interactions inferred from structure-based alignment of FZC18, SFRP3 and FZ8 CRDs and from described mutations affecting Wnt-CRD binding [25]. Red, localization of the RH3 peptide. Yellow, red and green overlay. 3D structure prediction was done using the Phyre www server and Protein Explorer 2.79. (1.74 MB TIF) [file pone.0001878.s003.tif]

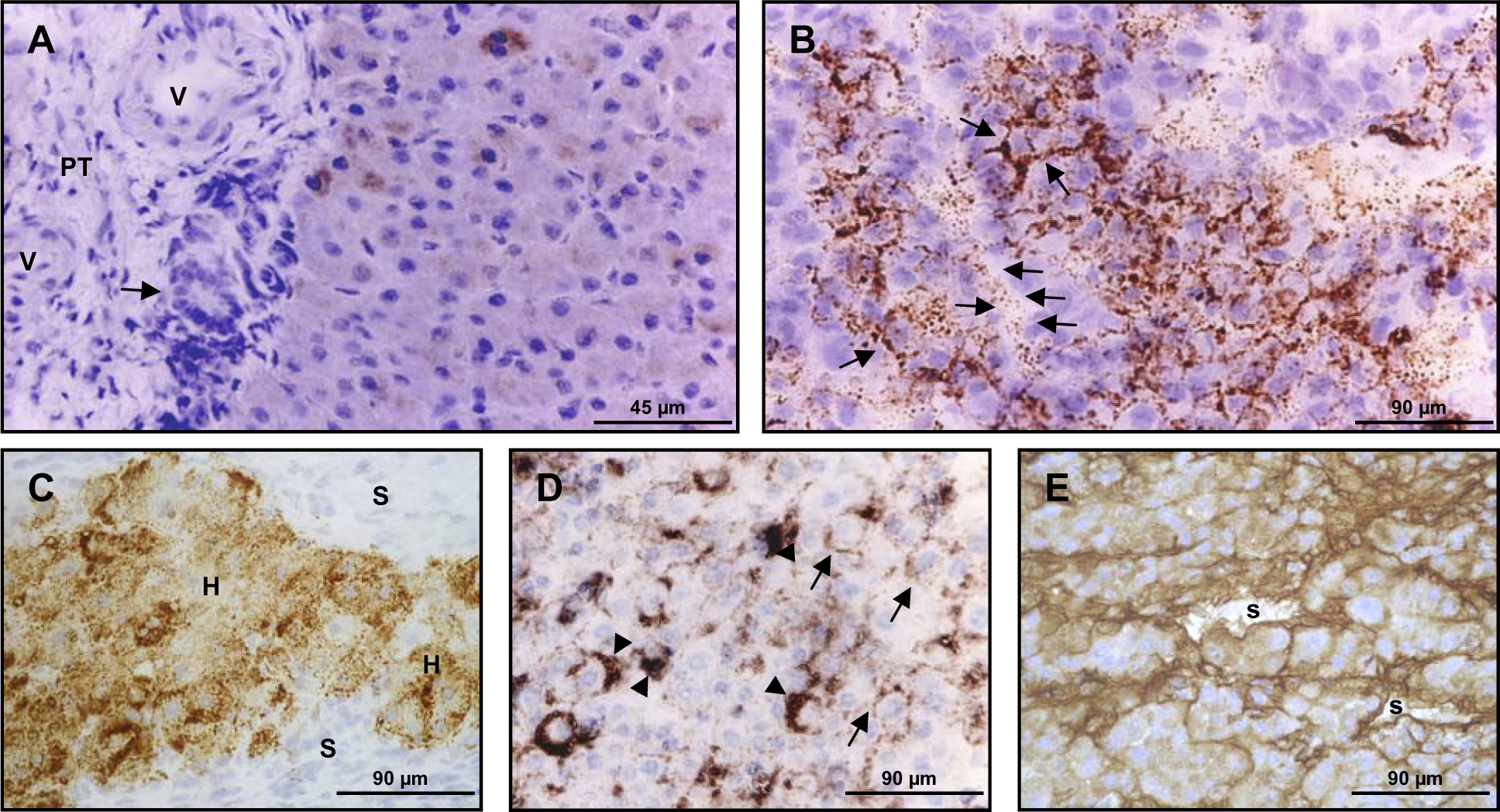

Supplement: Figure S4 — Detection of FZC18 in human liver. (A) Immunoperoxidase staining (brown) in normal human liver shows FZC18 in periportal hepatocytes. In the portal tract (PT), including portal veins (v), bile ducts and basement membrane (arrow) no signal is detected. Case NL 04, shown in Figure 8B. (B) HCC. Speckle-like FZC18 (arrows) is seen on the surface of tumor cells highlighting intercellular spaces (case TL 03, shown in Figure 8B). (C) Liver cirrhosis shows speckle-like FZC18 in hepatocytes (H). No FZC18 is seen in the surrounding stroma (S). (D and E) Case TL 06, shown in Figure 8, B and D–G. Contrasting topology of FZC18 (D) and DUF-959 (E) in HCC. FZC18 highlights the pericellular matrix (arrows) and is also detected as supranuclear intracellular material (arrowheads). In the same tumor, DUF-959 outlines cords of tumor cells highlighting tumor sinusoids (s). (9.05 MB TIF) [file pone.0001878.s004.tif]
